# Body mass index and use and costs of primary care services among women aged 55–79 years in England: a cohort and linked data study

**DOI:** 10.1038/s41366-018-0288-6

**Published:** 2018-12-19

**Authors:** Seamus Kent, Susan A Jebb, Alastair Gray, Jane Green, Gillian Reeves, Valerie Beral, Borislava Mihaylova, Benjamin J Cairns, on behalf of the Million Women Study collaborators, Hayley Abbiss, Simon Abbott, Rupert Alison, Miranda Armstrong, Krys Baker, Angela Balkwill, Isobel Barnes, Valerie Beral, Judith Black, Roger Blanks, Kathryn Bradbury, Anna Brown, Benjamin Cairns, Dexter Canoy, Andrew Chadwick, Dave Ewart, Sarah Ewart, Lee Fletcher, Sarah Floud, Toral Gathani, Laura Gerrard, Adrian Goodill, Jane Green, Lynden Guiver, Alicia Heath, Darren Hogg, Michal Hozak, Isobel Lingard, Sau Wan Kan, Nicky Langston, Kath Moser, Kirstin Pirie, Alison Price, Gillian Reeves, Keith Shaw, Emma Sherman, Rachel Simpson, Helena Strange, Sian Sweetland, Sarah Tipper, Ruth Travis, Lyndsey Trickett, Anthony Webster, Clare Wotton, Lucy Wright, Owen Yang, Heather Young, Emily Banks, Valerie Beral, Lucy Carpenter, Carol Dezateux, Jane Green, Julietta Patnick, Richard Peto, Cathie Sudlow

**Affiliations:** 1 Health Economics Research Centre, Nuffield Department of Population Health, University of Oxford, Richard Doll Building, Old Road Campus, Oxford, OX3 7LF USA; 2 Nuffield Department of Primary Care Health Sciences, University of Oxford, Radcliffe Observatory Quarter, Woodstock Road, Oxford, OX2 6GG USA; 3 Cancer Epidemiology Unit, Nuffield Department of Population Health, University of Oxford, Richard Doll Building, Old Road Campus, Oxford, OX3 7LF USA; 4 Centre for Primary Care and Public Health, Barts and The London School of Medicine and Dentistry, Queen Mary University of London, Barts and The London School of Medicine and Dentistry, Yvonne Carter Building, London, E1 2AB USA; 5 Medical Research Council Population Health Research Unit, Clinical Trial Service Unit & Epidemiological Studies Unit, Nuffield Department of Population Health, University of Oxford, Big Data Institute, Old Road Campus, Oxford, OX3 7LF USA

**Keywords:** Health policy, Public health

## Abstract

**Background:**

Excess weight is associated with poor health and increased healthcare costs. There are no reliable data describing the association between BMI and the use and costs of primary care services in the United Kingdom.

**Methods:**

Among 69,440 participants in the Million Women Study with primary care records in the Clinical Practice Research Datalink between April 2006 (mean age 64 years) and March 2014, the annual rates and costs of their primary care consultations, prescription medications, and diagnostic and monitoring tests were estimated in relation to their self-reported body mass index (BMI) at recruitment in 1996–2001 (mean age 56 years). Associations of BMI with annual costs were projected to all women in England aged 55–79 years in 2013.

**Results:**

Over an average follow-up of 6.0 years, annual rates and mean costs were lowest for women with a BMI of 20 to <22.5 kg/m^2^ for consultations (7.0 consultations, 99% CI 6.8–7.1; £288, £280–£295) and prescription medications (27.0 prescribed items, 26.0–27.9; £227, £216–£237). Above 20 kg/m^2^, a 2 kg/m^2^ higher BMI (a 5 kg change in weight for a woman of average height) was associated with 5.2% (4.8–5.6) and 9.9% (9.2–10.6) higher mean annual consultation and prescription medication costs, respectively. Annual rates and mean costs of diagnostic and monitoring tests were similar for women with different BMIs. Among all women aged 55–79 years in England, excess weight accounted for an estimated 11% (£229 million/£2.2 billion) of all consultation costs and 20% (£384 million/£1.9 billion) of all prescription medication costs, of which 27% were for diabetes drugs, 19% for circulatory system drugs, and 13% for analgesics.

**Conclusions:**

Excess body weight is associated with higher use and costs of primary care services among women in England. Reducing the prevalence of excess weight could improve the health of women and reduce pressures on primary care.

## Introduction

The prevalence of overweight and obesity (body mass index [BMI] ≥25 kg/m^2^) has increased substantially in many countries in recent decades. Between 1975 and 2015 its prevalence among adults in the United Kingdom (UK) increased from 45 to 68% in men and from 33 to 58% in women [1]. Excess weight is associated with an increased incidence of conditions, including type-2 diabetes, vascular diseases, osteoarthritis, depression, and certain cancers, as well as with higher mortality [2–4].

A recent systematic literature review reported that overweight and obesity were associated with higher costs for major healthcare services, with the greatest relative increase for medications, followed by inpatient care, and then ambulatory care [5]. Using data on over one million women in the UK, higher BMI was shown to be strongly associated with higher annual rates and costs of hospital admissions, overall and for a range of health conditions [6]. However, previous studies of primary healthcare services have generally been based on small-to-moderate numbers of participants, and are mostly from the United States (US), with no reliable evidence from the UK. It is unclear how well these results translate to the UK given the large differences in medication prices [7], and the role of primary healthcare in the UK in determining access to most specialist care [8].

Using individual participant data from a large cohort of women in England linked to routinely collected primary care records, we describe and quantify the relationship between body mass index and the use and costs of primary care services. This also provides insights into how much of the morbidity experienced by this group of women is potentially avoidable.

## Subjects and methods

### Million women Study

Between 1996 and 2001, 1.25 million women in England and 120,000 in Scotland aged 50–64 years were recruited into the million women study (MWS) through National Health Service (NHS) breast screening centres. Women who were invited to breast screening and to join the MWS were sent a study questionnaire with their invitation, which included questions about anthropometric, demographic, health, and other personal characteristics. In total around one-quarter of UK women in the eligible age range during recruitment participated in the study. Information on data access for the MWS is available at the study website.

### Linkage to health records

For women recruited in England, information was sought on death, emigration, and cancer registrations from NHS Central Registers, hospital admissions from Hospital Episode Statistics, and primary care records from the Clinical Practice Research Datalink (CPRD). Participants were linked to medical records using their unique NHS identification number, age, sex, and postcode.

CPRD collates longitudinal primary care medical records for around 7% of the UK population. Clinical data are recorded using version-2 Read codes, a hierarchical clinical classification system used in the UK [9], and prescriptions by the product name and British National Formulary (BNF) paragraph code, which provides information on prescribing and pharmacology for medications available on the UK NHS [10].

Information for 101,836 participants in the MWS were successfully linked to the CPRD GOLD database, and their primary care records up to January 2014 were extracted. These participants were largely similar to the other participants not registered in CPRD-participating practices (Table S1). For the purpose of the present analysis, we excluded: 4 women with unknown vital status; 6503 women whose primary care records were flagged as of poor quality by CPRD (83% of whom had only a temporary registration in the practice); 2133 women with registration gaps in their medical records; and, 6073 women whose follow-up in CPRD ended before recruitment into the MWS or before the date on which the data from their general practitioner’s practice was judged to be of up-to-standard quality according to CPRD.

In our main analysis, we also excluded: 1473 women with a recorded diagnosis of cancer (other than non-melanoma skin cancer) before recruitment; 4560 women for whom height or weight data were not available from the MWS recruitment questionnaire; 857 women with a BMI < 18.5 kg/m^2^; and 10,793 women for whom no data were recorded in CPRD beyond March 2006. Follow-up data prior to April 2006 were excluded from the main analysis because of the major expansion of the NHS’s primary care Quality and Outcomes Framework (QOF) in April 2006, which may have influenced clinical practice [8]. Following these exclusions, 69,440 women contributed person-years of data to analyses from April 2006 (or the date from which the practice to which they belonged was considered up-to-standard by CPRD, if later), until the earliest of their date of death, emigration, or 27 January 2014, the last date of CPRD data available for the present study.

### Ethical approval

Ethical approval for the MWS was provided by the Oxford and Anglia Multi-Centre Research Ethics Committee and participants gave signed consent for follow-up through their medical records. The use of CPRD data for this study was approved by the CPRD Independent Scientific Advisory Committee.

### Categorisation and costing of primary care services

We included all consultation records for the participants relating to a face-to-face surgery or clinic visit, home visit, out-of-hours visit, or telephone consultation, performed by a general practitioner (GP; a primary care doctor), a nurse, or allied health or social care professional (Table S2). Monitoring and diagnostic tests that are routinely performed as part of a standard consultation (e.g. lung capacity or blood pressure tests) were excluded as their costs are included in the costs of consultations. Average NHS costs (in 2016 prices) were applied to other categories of consultations (Table S3) and tests (Table S4) [11, 12].

Average costs per prescription item at the BNF paragraph level (Table S5) were calculated from the 2016 NHS Prescription Cost Analysis [13], and were applied to each prescription item issued in CPRD based on the BNF paragraph recorded by the GP. Therapies with unrecognised BNF codes in CPRD largely relate to devices and appliances, and an average cost across all such codes was applied.

Each prescription item was uniquely allocated to one of 18 categories of ‘therapeutic use’ corresponding to each of the 15 standard BNF chapters, plus analgesics, drugs in diabetes, and dressings and appliances. For medications of the circulatory system, medication use was further categorised by BNF section (e.g. lipid-regulating drugs).

### Statistical analysis

Rates and mean annual costs of consultations, tests, and prescribed items, were estimated by BMI category at recruitment (18.5 to <20, 20 to <22.5, 22.5 to <25, 25 to <27.5, 27.5 to <30, 30 to <35, 35 to <40, and 40 kg/m^2^ or more) [14]. Percentage differences in rates and mean annual costs per 2 kg/m^2^ higher BMI (a change in weight of ~5 kg for a woman of average height [162 cm] in England) were calculated for women with a BMI above 20 kg/m^2^, both overall and, in the case of prescriptions, by category of therapeutic use. All models were estimated using quasi-likelihood generalised linear models with a log-link and Poisson-like variance allowing for overdispersion.

In all models, further adjustments were made for age (in 5-year bands), region of recruitment, socioeconomic status [15], parity, age at birth of first child, smoking, alcohol intake, educational qualifications, financial year, and the proportion of each year with contributed data. Missing values for any of the adjustment variables (≤5% for all variables) were assigned to a separate category for that variable.

Cluster-robust standard errors were estimated in all models to account for the lack of independence between outcomes for a given individual across years of follow-up. To facilitate comparisons between any two BMI categories, even when neither is the reference category, group-specific 99% CIs were derived from the variance of the logarithm of the relative risks in each category [16].

Estimates of mean annual rates and costs were standardised to the participants contributing to the analysis based on the variables controlled for in regression. Annual costs were projected to the whole population of 6.6 million women aged 55–79 years in England in 2013 using the data on distribution of women by self-reported BMI from the Health Survey for England [17, 18]. Ninety-nine percent of confidence intervals were estimated by randomly simulating model parameters 10,000 times (based on the point and variance estimates from the MWS), and applying the bootstrap percentile method to generate the confidence interval limits.

We conducted sensitivity analyses to test the associations between the BMI of women and annual costs of consultations, tests, and prescriptions were estimated; including women with a history of cancer at baseline; including all years of follow-up from recruitment; excluding women with BMI >50 kg/m^2^; restricting the analysis to never-smokers; and excluding participants with self-reported heart disease or stroke at recruitment. Estimates of percentage differences in annual costs per 2 kg/m^2^ higher BMI were additionally estimated after replacing self-reported BMI from the MWS with the mean measured values of BMI within each category of self-reported BMI from the Health Survey for England (Table S6) [17].

Annual costs were also estimated within subgroups of women defined by age at the start of each annual period, and by smoking status, alcohol intake, strenuous exercise, socioeconomic status, and educational qualifications at recruitment. Heterogeneity of proportional increases in annual costs between categories of each subgroup was assessed using a *χ*^2^-test.

An exploratory analysis was undertaken to study the extent to which the primary care costs attributable to excess weight might be explained by diabetes. This is given as the difference between the estimated proportion of excess weight-attributable costs from the primary analysis and that derived from a model with diabetes status added as a covariate. Diabetes was identified using self-reported diabetes status in the MWS, and primary care and hospital records (Statistical Appendix). A woman was deemed to have diabetes in the annual period in which evidence for diabetes was first encountered and in all subsequent years.

All analyses were conducted using R 3.3.3. Further details of methods are available in the statistical appendix. Computer code is available from the corresponding author upon request.

## Results

The 69,440 women included in the main analysis were followed in CPRD for an average of 6.0 years from April 2006 (11.1 years from recruitment into the MWS) [Table 1]. Their mean age was 56.0 years (SD 4.8) at recruitment and 64.2 (SD 5.2) years at the start of the analysis period (April 2006 or later). At recruitment 47% of women had a BMI < 25kg/m^2^, 36% were overweight (BMI 25 to ≤30 kg/m^2^), and 17% were obese (BMI ≥30 kg/m^2^). Women who were overweight or obese tended to be of lower socioeconomic status and were less likely to do any strenuous exercise, drink alcohol, or be current smokers, but were more likely to be former smokers.

**Table 1:** Baseline characteristics and details of follow-up by category of body mass index

	Body mass index (kg/m^2^) at recruitment into MWS								
	18.5 to <20	20 to <22.5	22.5 to <25	25 to <27.5	27.5 to <30	30 to <35	35 to <40	≥40	All women
Number of women	1950	11,254	19,303	14,996	9866	8591	2575	905	69,440
*Characteristics at recruitment into MWS*									
Body mass index, median (IQR)	19.4 (0.6)	21.5 (1.1)	23.8 (1.3)	26.1 (1.3)	28.6 (1.2)	31.8 (2.3)	36.6 (2.3)	42.3 (3.9)	25.3 (5.4)
Age, mean (SD)	55.8 (4.8)	55.6 (4.8)	55.9 (4.8)	56.3 (4.8)	56.3 (4.8)	56.3 (4.8)	55.8 (4.6)	55.7 (4.6)	56.0 (4.8)
*Deprivation third in study population (%)*									
Least deprived	35.5	39.8	39.3	36.0	34.2	30.1	27.7	23.5	36.1
Most deprived	31.8	26.7	26.7	30.1	32.6	36.3	42.4	47.5	30.5
*Educational qualifications (%)*									
No qualifications	36.0	33.5	38.1	42.7	46.8	50.6	53.6	57.1	41.9
Secondary or technical	45.4	48.6	47.0	44.5	41.6	39.4	38.3	34.7	44.5
Tertiary	18.5	17.8	14.9	12.8	11.6	10.0	8.0	8.2	13.6
*Smoking status (%)*									
Never	51.0	52.8	53.4	52.2	51.5	51.2	52.8	49.2	52.4
Former	19.8	25.3	27.9	29.1	31.1	32.9	34.1	37.7	28.9
Current	29.2	21.9	18.7	18.7	17.4	15.9	13.1	13.1	18.7
Current alcohol drinkers (%)	75.5	81.2	81.8	78.8	75.0	71.1	63.1	57.6	77.6
Exercise rarely or never (%)	17.3	14.4	15.8	19.1	23.5	28.9	33.1	41.6	20.0
With prior health conditions (%)^a^	21.2	18.8	20.5	24.3	28.3	33.7	39.7	46.5	24.9
*Details of follow-up in CPRD from 1 April 2006*									
Age^b^, mean (SD)	64.1 (5.2)	63.8 (5.1)	64.1 (5.2)	64.4 (5.2)	64.4 (5.2)	64.4 (5.1)	63.9 (5.0)	63.7 (5.0)	64.2 (5.2)
Years of follow-up (1000s)	11.3	66.9	115.6	89.7	59.0	51.4	15.1	5.2	414.2
Total number of consultations (1000s)	76	434	780	650	466	453	149	57	3069
Total number of prescriptions items issued (1000s)	309	1631	3144	2926	2296	2479	881	383	14,051
Total number of tests (1000s)	100	570	996	832	547	478	141	56	3723

Percentages exclude participants with missing data on characteristics; percentage of missing data is less than 3% for all characteristics except for smoking status (5%)

*MWS* million women study, *IQR* interquartile range, *SD* standard deviation, *CPRD* clinical practice research datalink

^a^Any of self-reported heart disease, stroke, diabetes, rheumatoid arthritis, osteoarthritis, osteoporosis, or depression/anxiety

^b^Age at the start of the analysis period, i.e. 1 April 2006 or, if later, the date from which their primary care practice provided data of sufficient quality for research

**Fig. 1:**
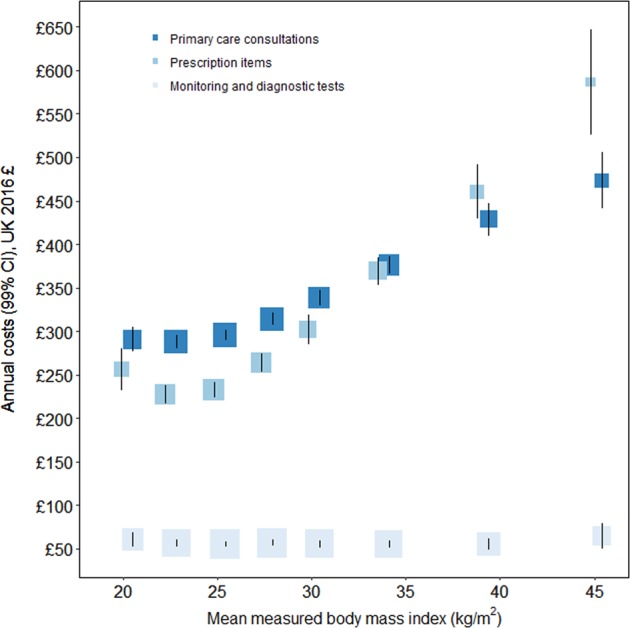
Annual primary care consultation, test, and prescription costs per person by category of body mass index. The standardised estimates of mean annual costs (in UK 2016 prices) are adjusted for age, region of recruitment, deprivation, educational qualifications, parity, age at birth of first child, smoking, alcohol intake, financial year, and proportion of year with contributed data. Annual costs are plotted against mean measured BMI (with a small offset to avoid overlaid CIs) within categories of self-reported BMI from the combined 2012 and 2013 Health Surveys for England (Table S6). The area of each square is inversely proportional to the variance of that estimate. The error bars show 99% CIs

Mean rates and mean annual costs of consultations were lowest for women with BMI 20 to < 22.5 kg/m^2^, at 7.0 consultations per year (99% CI 6.8–7.1) and £288 per year (280–295), respectively. Women with a BMI of ≥40 kg/m^2^ had an average of 11.1 consultations per year (10.3–11.9) at a mean cost of £473 per year (441–506) [Table 2, Fig. 1]. Mean annual rates and costs of prescription medications were also lowest for women with BMI 20 to <22.5 kg/m^2^ at 27.0 prescribed items per year (26.0–27.9) and £227 per year (216–237), respectively, and rose to 69.2 items (63.6–74.8) and £587 (525–648) for women with BMI ≥40 kg/m^2^. Participants had on average 8.4 diagnostic or monitoring tests per year (standard deviation [SD] 23.0) at a cost of £53 per year (SD 166), but there was no evidence of an association with BMI. For each 2 kg/m^2^ higher BMI above 20 kg/m^2^, the annual consultation and prescription costs for women were 5.2% (4.8–5.6) and 9.9% (9.2–10.6) higher, respectively. Annual prescription costs were elevated among women with BMI of 18.5 to <20 kg/m^2^ (£256 per year, 231–280) compared to BMI 20 to <22.5 kg/m^2^, but consultation and test costs were similar.

**Table 2:** Annual rates and costs of consultations, tests, and prescription items issued, by body mass index

	Rate per person-year		Annual costs per person	
BMI category (kg/m^2^)	Number per year	Difference in rate (%)^a^	Annual costs (2016 UK £)	Difference in costs (%)^a^
*Primary care consultations*				
18.5 to <20	7.0 (6.7, 7.3)	0.4% (−4.2, 5.2)	£290 (276, 304)	0.9% (−3.8, 5.8)
20 to <22.5 (reference)	7.0 (6.8, 7.1)	0.0% (−2.0, 2.0)	£288 (280, 295)	0.0% (−2.0, 2.1)
22.5 to <25	7.1 (7.0, 7.3)	2.7% (1.1, 4.2)	£296 (289, 302)	2.7% (1.2, 4.3)
25 to <27.5	7.5 (7.4, 7.7)	8.4% (6.6, 10.1)	£314 (307, 321)	9.1% (7.3, 10.9)
27.5 to <30	8.1 (7.9, 8.3)	16.7% (14.4, 19.0)	£338 (329, 347)	17.5% (15.1, 19.8)
30 to <35	9.0 (8.7, 9.2)	28.8% (26.1, 31.6)	£376 (366, 386)	30.7% (27.8, 33.5)
35 to <40	10.1 (9.7, 10.5)	45.0% (39.3, 50.9)	£428 (410, 447)	48.9% (42.9, 55.3)
≥40	11.1 (10.3, 11.9)	59.2% (48.9, 70.3)	£473 (441, 506)	64.5% (53.5, 76.3)
*Diagnostic tests*				
18.5 to <20	8.3 (7.2, 9.4)	3.3% (−9.5, 18.0)	£60 (52, 68)	6.7% (−6.0, 21.1)
20 to <22.5 (reference)	8.0 (7.5, 8.5)	0.0% (−5.8, 6.1)	£56 (52, 60)	0.0% (−5.4, 5.8)
22.5 to <25	8.1 (7.6, 8.5)	0.9% (−3.2, 5.2)	£55 (52, 58)	−2.0% (−6.0, 2.1)
25 to <27.5	8.6 (8.1, 9.1)	7.3% (2.4, 12.4)	£56 (53, 60)	0.4% (−4.2, 5.2)
27.5 to <30	8.6 (8.0, 9.1)	7.3% (1.3, 13.6)	£55 (51, 59)	−1.8% (−7.4, 4.1)
30 to <35	8.6 (8.0, 9.2)	7.0% (0.7, 13.8)	£55 (50, 59)	−2.6% (−8.6, 3.9)
35 to <40	8.6 (7.6, 9.6)	7.8% (−3.3, 20.2)	£55 (48, 62)	−2.4% (−13.1, 9.6)
≥40	10.0 (7.9, 12.2)	25.5% (1.3, 55.4)	£64 (50, 78)	14.4% (−8.0, 42.1)
*Prescription items issued*				
18.5 to <20	28.4 (26.2, 30.6)	5.4% (−2.2, 13.5)	£256 (231, 280)	12.9% (2.5, 24.3)
20 to <22.5 (reference)	27.0 (26.0, 27.9)	0.0% (−3.0, 3.1)	£227 (216, 237)	0.0% (−4.0, 4.2)
22.5 to <25	29.3 (28.5, 30.1)	8.7% (6.4, 11.1)	£233 (224, 241)	2.6% (−0.6, 5.8)
25 to <27.5	33.8 (32.8, 34.8)	25.4% (22.5, 28.3)	£264 (253, 274)	16.4% (12.7, 20.2)
27.5 to <30	39.2 (37.9, 40.5)	45.3% (41.4, 49.4)	£302 (285, 318)	33.1% (26.8, 39.7)
30 to <35	47.4 (45.9, 49.0)	76.0% (71.1, 81.0)	£369 (353, 385)	62.8% (56.7, 69.1)
35 to <40	57.0 (53.9, 60.1)	111.5% (100.4, 123.2)	£460 (429, 492)	103.0% (90.1, 116.9)
≥40	69.2 (63.6, 74.8)	156.5% (136.5, 178.3)	£587 (525, 648)	158.7% (134.1, 185.9)

All models are adjusted for age, region of recruitment, deprivation, educational qualifications, parity, age at first birth, smoking, alcohol intake, financial year, and proportion of year with contributed data. Values are means (99% confidence intervals)

*BMI* body mass index

^a^Differences are presented as percentage differences compared to BMI 20 to <22.5 kg/m^2^, with floating confidence intervals

Estimates of percentage differences in annual costs of primary care consultations, prescriptions, and diagnostic tests by BMI were not affected by the inclusion of women with previous cancer, the exclusion of women with BMI ≥50 kg/m^2^ or with previous heart disease or stroke, or when using imputed data to account for measurement error in BMI derived from self-reports (Tables S7-9). For annual consultation costs, estimates were also unaffected by the inclusion of outcome data prior to 1 April 2006 or restriction to women who had never smoked. The inclusion of outcome data prior to 1 April 2006 led to marginally smaller percentage differences in annual prescription costs for women with higher BMIs, while restriction to never smokers increased the estimated association.

The percentage differences in annual costs per 2 kg/m^2^ higher BMI above 20 kg/m^2^ were similar between subgroups of women defined by smoking behaviour, alcohol consumption, socioeconomic status, and education (Figures S1-3). There was some statistical heterogeneity between different age groups for both consultation and prescription costs, with somewhat smaller increases among older women, and, for prescription costs only, a somewhat smaller association for physically active individuals compared to the inactive.

Extrapolating from the MWS results to all 6.6 million women aged 55–79 years in England in 2013, total annual consultation and prescription costs were estimated to be £2.2 billion and £1.9 billion, respectively (Table 3). 11% (£229 million) of annual consultation costs and 20% (£384 million) of total annual prescription costs were attributable to excess weight. Of the total excess weight attributable annual consultation and prescription costs, around 30% were among women who were overweight but not obese, and 38% among those with grade 1 obesity (BMI 30 to <35kg/m^2^).

**Table 3:** Annual primary care consultation and prescription costs attributed to excess weight among women aged 55–79 years in England

Body mass index (kg/m^2^)	Number of women aged 55–79 in England (million)	Total annual costs (£ million)	Costs attributed to excess weight	
			Absolute annual costs (£ million) (99% CI)	Proportion costs attributed (%) (99% CI)
*Primary care consultations*				
<25	2.83	826	—	—
25–29.9	2.28	737	71 (60, 82)	10 (8, 11)
30–34.9	1.06	399	88 (80, 97)	22 (20, 24)
35–39.9	0.30	130	41 (36, 46)	32 (29, 34)
≥40	0.16	75	28 (24, 33)	38 (34, 42)
≥25 (all overweight and obesity)	3.80	1340	229 (210, 248)	17 (16, 18)
*Prescriptions items issued*				
<25	2.83	661	—	—
25–29.9	2.28	636	112 (91, 132)	18 (15, 20)
30–34.9	1.06	392	147 (133, 162)	38 (35, 40)
35–39.9	0.30	139	70 (61, 79)	50 (47, 53)
≥40	0.16	92	56 (48, 65)	61 (57, 64)
≥25 (all overweight and obesity)	3.80	1259	384 (352, 418)	31 (28, 33)

Estimates were derived by combining standardised estimates of annual costs per person (Table 2) and estimates of the number of women aged 55–79 in England by self-reported BMI category (Table S5). See further details of methods in statistical appendix

**Fig. 2:**
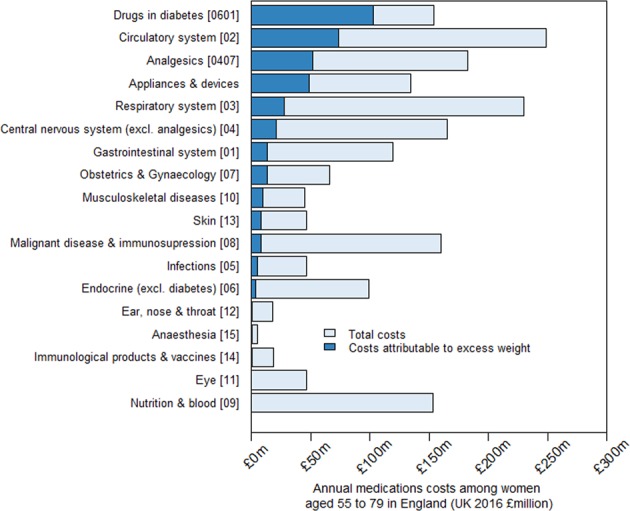
Annual prescription costs attributable to excess weight among women aged 55–79 years in England, by category of therapeutic use. Medications were categorised by therapeutic use (defined by BNF chapters or sections), and ordered here according to their contribution to overweight and obesity attributable costs. These estimates were derived by applying the estimates of excess costs by BMI category for each therapeutic use category from the Million Women Study analysis to women aged 55–79 in England using the Health Surveys for England 2012 and 2013 to estimate the population level distribution of women by self-reported BMI category and ONS mid-2013 population estimates. Excess costs were calculated relative to a BMI category of 20–24.9, estimated as a weighted average of the estimates of the two sub-categories (20 to <22.5 and 22.5 to <25)

Excess weight was associated with higher prescription costs for most categories of therapeutic use (Fig. 2; Figure S4; Table S10). Of the £384 million annual prescription medication costs attributed to excess weight among women aged 55–79 years in England, £102 million (27% of costs attributable to excess weight) was for drugs in diabetes, £73 million (19%) for circulatory system medications, and £51 million (13%) for analgesics. Drugs for hypertension and heart failure (£14 million), anticoagulants and protamine (£17 million), and lipid-regulation (£15 million), each accounted for around 20% of the excess weight-attributable costs for the circulatory system.

Diabetes was self-reported at recruitment into the study by 1692 participants (3%). By the end of follow-up, and as further indicated in primary and secondary healthcare data, 8226 (12%) had some evidence of diabetes. We estimated that diabetes was associated with 37% of consultation costs and 47% of prescription costs attributed to excess weight.

## Discussion

This analysis demonstrates the impact of excess weight on healthcare use by women aged over 50 years in the UK. Higher BMI is associated with higher annual rates and costs of primary care consultations and prescription medications, but not diagnostic and monitoring tests. Among all women in England aged 55–79 years, excess weight accounted for 11% of total annual consultation costs and 20% of annual prescription costs, of which 27% were for diabetes drugs, 19% for circulatory system drugs, and 13% for analgesics.

A recent systematic review identified 18 individual participant data studies that estimated prescription medication costs in relation to BMI, with sample sizes ranging from 2244 to 17,703 [5]. Compared to adults with healthy weight, annual costs were, on average, 18 and 64% higher for overweight and obese adults, respectively. The corresponding estimates in our study are somewhat larger, at 23 and 79%. In the MWS, analgesics and drugs for diabetes and circulatory disease accounted for about 30% of all prescription costs, but almost 60% of the costs attributable to excess weight, with drugs for diabetes the largest contributor. Few other studies estimated medication costs in relation to BMI for different therapeutic uses; those that did also tended to find the strongest proportional effects of high BMI on costs for diabetes and cardiovascular medications, and analgesics, but with cardiovascular medications contributing the greatest proportion of excess costs associated with overweight and obesity [19–23]. The differences in estimates in our study compared to previous studies, which were based mainly on US populations, are likely to reflect an older average age of participants in the MWS, and differences in healthcare systems, with varying accessibility of healthcare, clinical practice, and prices for medications [7, 24].

The lack of association between diagnostic and monitoring test costs and excess weight is surprising and has not been previously reported. Given the greater prevalence of ill health and frequency of consultations among women who are overweight or obese, one interpretation is that these women are proportionally less likely to be offered diagnostic tests. This is a concern and warrants further investigation as it may lead to delayed diagnosis and exacerbate avoidable morbidity.

Few studies have estimated primary care consultation costs in relation to BMI and previous studies were based on small sample sizes (500–3000 participants) [23, 25–29]. Estimates of the relative costs associated with obesity compared to healthy weight varied greatly from a 25% reduction [25] to a 160% increase [26]. Again, most studies used data from the US, where the primary care system differs substantially from that in the UK [8]. The few studies that reported costs separately for diagnostic tests reported marginally higher costs with higher BMI [25, 30].

Evidence of the associations between primary care costs and BMI were mostly similar in population subgroups. There was some evidence of weaker associations among older adults, with each unit higher BMI associated with 45 and 36% higher annual consultation and prescription costs, respectively, in women aged less than 65 years compared to women 70 years or older. This is consistent with previous studies of associations between BMI and mortality and hospital admissions [3, 31]. This could be a result of changes to body composition in older adults, who tend to have less fat-free mass, or a consequence of reverse causality due to higher rates of comorbidities in older adults [32]. Associations were also about 20% smaller for physically active adults compared to inactive adults for prescription costs, although no difference was observed for consultation costs. Differences in healthcare use associated with excess weight by level of physical activity could arise because physical activity offsets some of the adverse health effects of excess weight or because of preferences for lifestyle modifications over pharmacological treatment for conditions like diabetes or cardiovascular disease, but these hypotheses cannot be conclusively assessed in these data.

The projection of results from this study to all women aged 55–79 years in England assumes that the distribution of characteristics within BMI categories is similar among women in the general population and the study population. Study participants were representative of women attending breast cancer screening during the recruitment period of the study [33], they were more likely to come from less deprived areas and to have a current prescription for hormone replacement therapy, but did not differ in terms of age or recent prescriptions for various other medications [34]. Differences between women who participated in the study and those who did not could result in a small bias to the estimated associations, but this would not be expected to substantially change our findings.

Our findings are based on observational data and we have made efforts to deal with confounding using statistical adjustment, and with reverse causality by excluding the first 5 years of follow-up after recruitment. However, residual biases may remain. BMI was derived from self-reported height and weight and may systematically underestimate true BMI [35], but BMI derived from self-reported height and weight in the MWS is closely correlated with BMI derived from measured height and weight nine years after recruitment, and is suitable to accurately estimate linear associations [36]. We also excluded women with missing height or weight. Although women with missing data may differ from women with complete data, the proportion of women excluded was small, and we would not expect their exclusion to make an appreciable difference to the estimated associations.

Estimates in this study reflect clinical practice in England during the period of study follow-up, including clinical decisions and prescription guidelines. However, clinical practice varies over time in response to a number of factors including new drugs and technologies, patent expirations, and new medical evidence [37]. In other populations or at other times, the estimated associations between BMI and costs might differ. Costs of prescription medications also do not include the dispensing fee paid to pharmacists or any savings to the NHS from wholesale purchase or special arrangements with manufacturers.

Our findings of higher primary care use and costs with higher BMI complement previous results from the MWS reporting higher hospital admissions rates and costs with higher BMI in middle aged and older women in England, and emphasise the impact of excess weight on the health of women [6]. Previous research has suggested that population ageing has contributed to the increasing workload in primary care, but there are also increases in age-sex standardised rates [38]. Our research suggests that rising rates of obesity are also likely to be an important contributor. The finding that two-thirds of excess weight attributable costs were incurred among women who were overweight or mildly obese (BMI < 35 kg/m^2^) makes a case for clearer signposting to treatment services or advice for self-management to all women with excess weight [39, 40]. Weight loss would benefit women through improved health and would be expected to decrease healthcare usage. The results should also be useful to healthcare commissioners and planners making investment and prioritisation decisions, particularly in relation to local needs and expectations of changes in overweight and obesity rates. Qualitative research which engages with women may reveal additional opportunities to enhance healthcare services for this group. Future research should also investigate the associations in men and in younger individuals, and seek to identify the contributions of different health conditions to the consultation costs that are attributable to excess weight.

## Supplementary information

Supplementary Materials
