# Supplementary material for: Body mass index and use and costs of primary care services among women aged 55–79 years in England: a cohort and linked data study
Source: Int J Obes (Lond). 2018 Dec 19;43(9):1839–48. doi: 10.1038/s41366-018-0288-6 (PMC6451629; doi:10.1038/s41366-018-0288-6)
Supplement: Supplementary file 1 — Supplementary Materials [file 41366_2018_288_MOESM1_ESM.docx]

**Supplementary Materials**

Table S1. Baseline characteristics of Million Women Study participants recruited in England with and without any records in CPRD

|  | | | **With data in CPRD?** | |
| --- | --- | --- | --- | --- |
|  | | | **No** | **Yes** |
| Number of women | | | 1,145,328 | 101,836 |
| Body mass index, mean SD) | | | 26.2 (4.7) | 26.1 (4.7) |
| Age at recruitment, mean (SD) | | | 56.2 (4.9) | 56.1 (4.8) |
| Deprivation tertile in study population (%) | | |  |  |
|  | Least deprived | | 33.1 | 35.1 |
|  | Most deprived | | 33.3 | 31.6 |
| Educational qualifications (%) | | |  |  |
|  | No qualifications | | 44.7 | 41.9 |
|  | Secondary or technical | | 42.7 | 44.0 |
|  | Tertiary | | 12.7 | 14.1 |
| Smoking status (%) | | |  |  |
|  | | Never | 51.3 | 51.2 |
|  | | Former | 28.5 | 29.0 |
|  | | Current | 20.2 | 19.8 |
| Current alcohol drinkers (%) | | | 76.0 | 76.9 |
| Exercise rarely or never (%) | | | 48.8 | 47.1 |
| With prior health conditions (%) | | | 26.2 | 26.1 |

Table S2. Categorisation of consultations by staff role and consultation type

|  | Category (frequency per 1,000 person-years) |
| --- | --- |
| **Staff role** |  |
| General practitioner | Senior Partner (3663); Sole Practitioner (2194); Commercial Deputising service (320); Locum (238); Partner (55); GP Retainer (52); Assistant (19); GP Registrar (1); Consultant (14); Community Medical Officer (2); Non-commercial local rota of less than 10 GPs (3) |
| Nurse or allied healthcare or social care professional | Pharmacist (53); Carer (351); Non-qualified Dispenser (47); Stomatherapist (53); Health Visitor (139); Dispenser (9); Social Worker (36); Speech Therapist (62); Other Additional Clinical Services (20); Chiropractor (17); Community Psychiatric Nurse (4); Physiotherapist (17); Dietician (6); Osteopath (1); School Nurse (12); Dentist (5); Practice Nurse (3); Other Health Care Professional (4); Other Healthcare Scientists (2); Clinical Practitioner Access Role (3); Midwife (1); Other Medical & Dental (1); Phlebotomist (1) |
| **Consultation type** |  |
| Surgery or clinic | Surgery consultation (5663); Third Party Consultation (171); Clinic (717); Medicine Management (104); Triage (96); Acute visit (29); Emergency Consultation (24); Casualty Attendance (3); Follow-up/routine visit (15) |
| Home visit or out-of-hours | Out of hours, Non Practice (31); Home Visit (70); Nursing Home Visit (4); Residential Home Visit (3) |
| Telephone | Telephone call from a patient (191); Telephone call to a patient (286); Co-op Telephone advice (1) |
| Frequencies calculated for women included in the main analysis from 1 April 2006. Categories of staff role and consultation type not observed in the data used here are not reported. | |

Table S3. Mean costs (UK 2016 prices) per consultation, by staff role and consultation type

| **Staff roles^a^** | **Consultation type** | **Cost** | **Description** |
| --- | --- | --- | --- |
| Senior Partner, Sole Practitioner, Commercial Deputising service, Locum; Partner, GP Retainer, Assistant, GP Registrar, Consultant, Community Medical Officer, Non-commercial local rota of less than 10 GPs | Surgery | £42.1 | Cost per minute GP time: PSSRU 2016 (Table 10.3b). Average consultation length of 11.7 minutes: 2006-7 GP workload survey. |
|  | Clinic | £61.9 | Cost per minute GP time: PSSRU 2016 (Table 10.3b). Average consultation length of 17.2 minutes: 2006-7 GP workload survey. |
|  | Home visit | £84.2 | Cost per minute GP time: PSSRU 2016 (Table 10.3b). Average consultation length of 11.4 minutes: 2006-07 GP workload survey; assume average 12 minutes travel time for home visits: PSSRU 2009. |
|  | Out-of-hours | £41.0 | Cost per minute GP time: PSSRU 2016 (Table 10.3b). Average consultation length of 11.4 minutes: 2006-7 GP workload survey. |
|  | Telephone | £25.6 | Cost per minute GP time: PSSRU 2016 (Table 10.3b). Average consultation length of 7.1 minutes: 2006-7 GP workload survey. |
| Practice Nurse, Pharmacist, Dispenser, Non-qualified Dispenser, Other Health Care Professional, School Nurse, Stomatherapist, Phlebotomist, Other Healthcare Scientists, Other Additional Clinical Services, Clinical Practitioner Access Role | Surgery, telephone | £14.7 | PSSRU 2015, Table 10.6 (practice nurse). Mean hourly cost is £57 for face-to-face contact. Average consultation is 15.5 minutes: 2006-7 GP workload survey. |
|  | Home visit | £19.4 | As above, but including 12 minutes travel time. |
| Dentist, Other Medical & Dental | Surgery | £130 | PSSRU 2016, Tables 10.5 (NHS performer only dentist) & 10.6 (providing-performer dentist). Weighted average of costs for hour long appointment by dentist type. |
| Health visitor, Midwife | All categories | £54.7 | PSSRU 2015, Table 10.3 (health visitor). |
| Community psychiatric nurse | All categories | £135 | NHS Reference Costs 2013/14, inflated to 2015/16 using HCHS pay and prices index. Also reported in PSSRU 2016 (Table 12.1). Given as mean average weighted cost per face-to-face contact for all community health teams for older people with mental health problems; 43% of these staff were community nurses. |
| Carer | All categories | £12 | PSSRU 2016, Table 11.6 (home care worker). Mean hourly cost of all home care is £24. Mean duration is assumed to be 30 minutes. |
| Social worker | All categories | £79 | PSSRU 2016, Table 11.2 (social worker [adult services]). Cost per consultation not given; have assumed 1 hour. |
| Dietician | All categories | £81 | NHS Reference Costs 2015-16, currency code: A03. |
| Speech and language therapist | All categories | £88.5 | NHS Reference Costs 2015-16. Weighted average of group and one-on-one attendances (currency codes: A13A1 & A13AG). |
| Physiotherapist, Osteopath, chiropractor | All categories | £48.9 | NHS Reference Costs 2015-16. Weighted average of group and one-on-one attendances (currency codes: A08A1 & A08AG). |
| ^a^As defined in CPRD. Costs include qualification costs but do not include direct care staff costs. | | | |

Table S4. Unit costs per test in UK 2016 prices (and frequency per 1,000 person-years)

| **Test** | **Unit cost (UK 2016 prices)** | **Source** |
| --- | --- | --- |
| **Laboratory tests** |  |  |
| Biochemistry | £1.18 | Directly accessed pathology services |
| Haematology | £7.63 |  |
| Microbiology | £3.13 |  |
| Serology & Immunology | £6.42 |  |
| Other Pathology Tests | £3.10 |  |
|  |  |  |
| **Diagnostic imaging** |  |  |
| Barium enema | £144.96 | Diagnostic imaging, direct access; weighted average cost of currency code RD3 |
| X-rays: Abdominal x-ray; Ankle x-ray; Chest x-ray; Foot x-ray; Hand x-ray; Hip x-ray; Knee x-ray; Mammogram; Neck x-ray; Pelvis x-ray; Shoulder x-ray; Spine x-ray; Upper leg x-ray; Wrist x-ray | £30.26 | Directly accessed diagnostic services, direct access plain film (currency code DAPF) |
| Ultrasound: Abdominal ultra sound; General ultra sound scan; Pelvic ultra sound | £52.82 | Diagnostic imaging, direct access; weighted average cost of currency code RD4 |
| CAT scan | £107.52 | Diagnostic imaging, direct access; weighted average cost of currency code RD2 |
| MRI scan | £147.25 | Diagnostic imaging, direct access; weighted average cost of currency code RD0 |
| Other diagnostic imaging | £70.64 | Diagnostic imaging, direct access; weighted average cost of all codes |
| Bone density studies | £68.29 | Diagnostic imaging, direct access; currency code RD50Z |
|  |  |  |
| **Diagnostic tests** |  |  |
| Electrocardiogram | £88.00 | Directly accessed diagnostic services, currency code AA33C |
| Colonoscopy | £421.32 | Outpatient, FZ51Z |
| Sigmoidoscopy | £205.85 | Outpatient, FZ54Z |
| Bronchoscopy | £458.52 | Outpatient, DZ69A |
| Endoscopy | £495.97 | Outpatient, FZ42A |
| Echocardiogram | £88.83 | Directly accessed diagnostic services, currency code EY50Z |
| ECG ambulatory; ECG exercise | £40.35 | Directly accessed diagnostic services, currency code EY51Z |
| Doppler ultrasound peripheral pulse | £75.85 | Diagnostic imaging, direct access; currency code RD47Z |
| Frequencies calculated for women included in the main analysis from 1 April 2006. | | |

Table S5. Mean costs per prescription issued (in UK 2016 prices) by BNF chapter, section, and paragraph

| **BNF chapter** | **BNF section** | **BNF paragraph** |
| --- | --- | --- |
| Gastrointestinal system (£4.8) | Dyspep&Gastro-Oesophageal Reflux Disease (£6.1) | Antacids and simeticone (£17.1); Compound alginates and proprietary indigestion preparations (£5.6) |
|  | Antispasmod.&Other Drgs Alt.Gut Motility (£13.7) | No paragraphs |
|  | Antisecretory Drugs+Mucosal Protectants (£1.9) | H2-receptor antagonists (£2.1); Selective antimuscarinics (£99.0); Chelates and complexes (£97.2); Prostaglandin analogues (£11.4); Proton pump inhibitors (£1.9) |
|  | Acute Diarrhoea (£4.3) | Adsorbents and bulk-forming drugs (£1.3); Antimotility drugs (£4.3); Enkephalinase inhibitors (£12.4) |
|  | Chronic Bowel Disorders (£44.0) | Aminosalicylates (£42.0); Corticosteroids (£80.5); Drugs affecting the immune response (£NA); Food allergy (£70.7) |
|  | Laxatives (£5.0) | Bulk-forming laxatives (£4.7); Stimulant laxatives (£3.4); Faecal softeners^a^ (£12.0); Osmotic laxatives (£5.6); Bowel cleansing preparations (£13.8); Peripheral opioid-receptor antagonists^a^ (£194.5); Other drugs used in constipation (£55.5) |
|  | Local Prepn for Anal & Rectal Disorders (£7.7) | Soothing haemorrhoidal preparations (£9.3); Compound haemorrhoidal preparations with corticosteroids (£5.1); Rectal sclerosants (£6.3); Management of anal fissures (£40.6) |
|  | Stoma Care (£5.5) | No paragraphs |
|  | Drugs Affecting Intestinal Secretions (£50.5) | Drugs affecting biliary composition and flow (£45.7); Bile acid sequestrants (£NA); Aprotinin (£NA); Pancreatin (£53.1) |
| Cardiovascular System (£3.4) | Positive Inotropic Drugs (£3.0) | Cardiac glycosides (£3.0); Phosphodiesterase type-3 inhibitors (£240.2) |
|  | Diuretics (£1.6) | Thiazides and related diuretics (£1.5); Loop diuretics (£1.2); Potassium-sparing diuretics and aldosterone antagonists (£3.7); Potassium-sparing diuretics with other diuretics (£3.7); Osmotic diuretics (£11.7); Mercurial diuretics (£NA); Carbonic anhydrase inhibitors (£NA); Diuretics with potassium (£3.8) |
|  | Anti-Arrhythmic Drugs (£9.3) | Management of arrhythmias (£NA); Drugs for arrhythmias (£9.3) |
|  | Beta-Adrenoceptor Blocking Drugs (£1.8) | No paragraphs |
|  | Hypertension and Heart Failure (£2.1) | Vasodilator antihypertensive drugs (£17.3); Centrally acting antihypertensive drugs (£5.8); Adrenergic neurone blocking drugs (£NA); Alpha-adrenoceptor blocking drugs (£2.6); Drugs affecting the renin-angiotensin system (£2.0) |
|  | Nit,Calc Block & Other Antianginal Drugs (£4.0) | Nitrates (£4.8); Calcium-channel blockers (£3.3); Other antianginal drugs (£11.7); Peripheral vasodilators and related drugs (£9.8) |
|  | Sympathomimetics (£56.1) | Inotropic sympathomimetics (£1222.9); Vasoconstrictor sympathomimetics (£56.2); Cardiopulmonary resuscitation (£26.1) |
|  | Anticoagulants And Protamine (£18.3) | Parenteral anticoagulants (£110.3); Oral anticoagulants (£15.7); Protamine sulfate (£NA) |
|  | Antiplatelet Drugs (£2.0) | No paragraphs |
|  | Stable Angina, Acute/Crnry Synd&Fibrin (£1800.0) | Management of stable angina and acute coronary syndromes (£NA); Fibrinolytic drugs^a^ (£6.3) |
|  | Antifibrinolytic Drugs & Haemostatics (£6.0) | No paragraphs |
|  | Lipid-Regulating Drugs (£3.1) | No paragraphs |
|  | Local Sclerosants (£9.6) | No paragraphs |
| Respiratory System (£15.6) | Bronchodilators (£10.6) | Adrenoceptor agonists (£4.2); Antimuscarinic bronchodilators (£34.5); Theophylline (£3.4); Compound bronchodilator preparations (£33.7); Peak flow meters, inhaler devices and nebulisers (£NA) |
|  | Corticosteroids (Respiratory) (£33.7) | No paragraphs |
|  | Cromoglycate,Rel,Leukotriene Antagonists (£2.9) | Cromoglicate and related therapy (£39.0); Leukotriene receptor antagonists (£2.7); Phosphodiesterase type-4 inhibitors (£35.7) |
|  | Antihist, Hyposensit & Allergic Emergen (£4.1) | Antihistamines (£2.9); Allergen immunotherapy (£121.7); Allergic emergencies (£52.5) |
|  | Mucolytics (£15.4) | No paragraphs |
|  | Aromatic Inhalations (£1.9) | No paragraphs |
|  | Cough Preparations (£1.5) | Cough suppressants (£1.9); Demulcent and expectorant cough preparations^a^ (£3.2) |
|  | Systemic Nasal Decongestants (£3.0) | No paragraphs |
|  | Antifibrotics (£1022.8) | No paragraphs |
| Central Nervous System (£8.9) | Hypnotics And Anxiolytics (£4.3) | Hypnotics (£5.8); Anxiolytics (£2.2); Barbiturates (£100.4) |
|  | Drugs Used In Psychoses & Rel.Disorders (£8.3) | Antipsychotic drugs (£7.4); Antipsychotic depot injections (£64.5); Drugs used for mania and hypomania (£6.9) |
|  | Antidepressant Drugs (£4.1) | Tricyclic and related antidepressant drugs (£7.5); Monoamine-oxidase inhibitors (£138.6); Selective serotonin re-uptake inhibitors (£1.6); Other antidepressant drugs (£6.0) |
|  | CNS Stimulants and drugs used for ADHD (£43.2) | No paragraphs |
|  | Drugs used in the Treatment of Obesity (£22.1) | Anti-obesity drugs acting on the gastro-intestinal tract (£22.1); Centrally acting appetite suppressants (£2.0) |
|  | Obesity (£22.1) | Anti-obesity drugs acting on the gastro-intestinal tract (£22.1); Centrally acting appetite suppressants (£2.0) |
|  | Drugs Used In Nausea And Vertigo (£5.5) | No paragraphs |
|  | Analgesics (£7.8) | Non-opioid analgesics and compound analgesic preparations (£4.9); Opioid analgesics (£12.3); Neuropathic pain^a^ (£17.3); Antimigraine drugs (£11.5) |
|  | Antiepileptics (£22.4) | Control of the epilepsies (£22.1); Drugs used in status epilepticus (£123.9); Febrile convulsions (£NA) |
|  | Antiepileptic Drugs (£22.4) | Control of the epilepsies (£22.1); Drugs used in status epilepticus (£123.9); Febrile convulsions (£NA) |
|  | Drugs Used In Park'ism/Related Disorders (£25.2) | Dopaminergic drugs used in Parkinson's disease (£27.4); Antimuscarinic drugs used in parkinsonism (£16.3); Drugs used in essential tremor, chorea, tics, and related disorders (£41.5) |
|  | Drugs Used In Substance Dependence (£13.9) | Alcohol dependence (£25.9); Nicotine dependence (£28.9); Opioid dependence (£8.4) |
|  | Drugs for Dementia (£8.2) | No paragraphs |
|  | Dementia (£8.2) | No paragraphs |
| Infections (£5.0) | Antibacterial Drugs (£5.0) | Penicillins (£3.3); Cephalosporins, carbapenems, and other beta-lactams (£3.2); Tetracyclines (£5.3); Aminoglycosides (£298.5); Macrolides (£4.8); Clindamycin (£37.0); Some other antibacterials (£182.4); Sulfonamides and trimethoprim (£2.1); Antituberculosis drugs (£36.2); Antileprotic drugs (£72.8); Metronidazole and tinidazole (£3.8); Quinolones (£5.5); Urinary-tract infections (£13.4) |
|  | Antifungal Drugs (£5.3) | Triazole antifungals (£8.4); Imidazole antifungals (£369.7); Polyene antifungals (£2.3); Echinocandin antifungals (£196.1); Other antifungals (£5.2) |
|  | Antiviral Drugs (£11.8) | HIV infection^a^ (£94.9); Herpesvirus infections (£9.0); Viral hepatitis^a^ (£23.1); Influenza (£15.2); Respiratory syncytial virus^a^ (£16.0) |
|  | Antiprotozoal Drugs (£2.3) | Antimalarials (£2.1); Amoebicides (£85.7); Trichomonacides (£NA); Antigiardial drugs (£184.6); Leishmaniacides (£236.4); Trypanocides (£NA); Drugs for toxoplasmosis (£NA); Drugs for pneumocystis pneumonia (£604.5) |
|  | Anthelmintics (£2.9) | Drugs for threadworms (£1.4); Ascaricides^a^ (£1.4); Drugs for tapeworm infections (£196.8); Drugs for hookworms (£NA); Schistosomicides (£290.4); Filaricides (£221.0); Drugs for cutaneous larva migrans (£NA); Drugs for strongyloidiasis (£NA) |
| Endocrine system (£13.2) | Drugs Used In Diabetes (£19.0) | Insulins (£49.5); Antidiabetic drugs (£11.9); Diabetic ketoacidosis (£NA); Treatment of hypoglycaemia (£13.2); Treatment of diabetic nephropathy and neuropathy (£NA); Diagnostic and monitoring devices for diabetes mellitus (£26.0) |
|  | Thyroid And Antithyroid Drugs (£5.2) | Thyroid hormones (£4.2); Antithyroid drugs (£60.1) |
|  | Corticosteroids (Endocrine) (£14.7) | Replacement therapy (£28.5); Glucocorticoid therapy (£14.0) |
|  | Sex Hormones (£10.4) | Female sex hormones and their modulators (£13.7); Male sex hormones^a^ and antagonists (£42.3); Anabolic steroids^a^ (£49.8) |
|  | Hypothalamic&Pituitary Hormones&Antioest (£127.4) | Hypothalamic and anterior pituitary hormones and anti-oestrogens (£468.9); Posterior pituitary hormones and antagonists (£41.1) |
|  | Drugs Affecting Bone Metabolism (£3.0) | Calcitonin and parathyroid hormone^a^ (£154.5); Bisphosphonates and other drugs affecting bone metabolism (£2.9) |
|  | Other Endocrine Drugs (£63.9) | Bromocriptine and other dopaminergic drugs (£59.0); Drugs affecting gonadotrophins (£39.2); Cushing's Syndrome (£409.4); Somatomedins^a^ (£20.4) |
| Obstetrics, gynaecology, and urinary-tract disorders (£11.9) | Drugs Used In Obstetrics (£132.6) | Prostaglandins and oxytocics (£132.6); Mifepristone (£NA); Myometrial relaxants (£NA) |
|  | Treatment Of Vaginal & Vulval Conditions (£11.8) | Preparations for vaginal and vulval changes (£17.0); Vaginal and vulval infections (£6.0) |
|  | Contraceptives (£9.3) | Combined hormonal contraceptives (£7.7); Progestogen-only contraceptives (£10.9); Spermicidal contraceptives (£12.9); Contraceptive devices (£NA); Emergency contraception (£7.1) |
|  | Drugs For Genito-Urinary Disorders (£13.2) | Drugs for urinary retention (£5.9); Drugs for urinary frequency, enuresis, and incontinence (£20.7); Drugs used in urological pain (£10.2); Bladder instillations and urological surgery (£46.2); Drugs for erectile dysfunction (£13.2); Drugs for premature ejaculation (£36.3) |
| Malignant disease and immunosupression (£50.7) | Cytotoxic Drugs (£91.7) | Alkylating drugs (£76.1); Anthracyclines and other cytotoxic antibiotics (£210.2); Antimetabolites (£94.0); Vinca alkaloids and etoposide (£NA); Other antineoplastic drugs (£87.6) |
|  | Drugs Affecting The Immune Response (£46.1) | Antiproliferative immunosuppressants (£12.9); Corticosteroids and other immunosuppressants^a^ (£40.5); Anti-lymphocyte monoclonal antibodies (£436.6); Other immunomodulating drugs (£1442.8) |
|  | Sex Hormones & Antag In Malig Disease (£50.5) | Oestrogens (£136.5); Progestogens (£38.6); Androgens (£NA); Hormone antagonists (£50.1) |
| Nutrition and blood (£11.1) | Anaemias + Other Blood Disorders (£3.2) | Iron-deficiency anaemias (£3.8); Drugs used in megaloblastic anaemias (£2.1); Drugs used in hypoplastic, haemolytic, and renal anaemias (£312.1); Drugs used in platelet disorders (£520.1); G6PD deficiency (£NA); Drugs used in neutropenia (£464.8); Drugs used to mobilise stem cells (£NA) |
|  | Fluids And Electrolytes (£7.5) | Oral preparations for fluid and electrolyte imbalance (£7.3); Parenteral preparations for fluid and electrolyte imbalance (£8.2) |
|  | Intravenous Nutrition (£39.7) | No paragraphs |
|  | Oral Nutrition (£49.1) | Foods for special diets (£44.3); Enteral nutrition (£51.9) |
|  | Minerals (£22.6) | Calcium and magnesium (£28.7); Calcium and magnesium (£28.7); Phosphorus (£54.4); Fluoride (£16.0); Zinc (£14.5); Selenium (£71.7) |
|  | Vitamins (£4.8) | Vitamin A (£5.3); Vitamin B group (£4.5); Vitamin C (£25.1); Vitamin D (£4.6); Vitamin E (£34.0); Vitamin K (£48.3); Multivitamin preparations (£3.4) |
|  | Bitters And Tonics (£463.0) | No paragraphs |
|  | Metabolic Disorders (£896.6) | Drugs used in metabolic disorders (£896.6); Acute porphyrias (£NA) |
|  | Foods (£70.4) | No paragraphs |
|  | Compound Vit/Mineral Formulations (£7.5) | No paragraphs |
|  | Health Supplements (£35.0) | No paragraphs |
|  | Other Health Supplements (£10.7) | No paragraphs |
| Musculoskeletal and joint diseases (£6.1) | Drugs Used In Rheumatic Diseases & Gout (£5.9) | Non-steroidal anti-inflammatory drugs (£4.5); Corticosteroids (£4.7); Drugs that suppress the rheumatic disease process (£18.1); Gout and cytotoxic-induced hyperuricaemia (£2.5); Other drugs for rheumatic diseases (£20.2) |
|  | Drugs Used In Neuromuscular Disorders (£11.8) | Drugs that enhance neuromuscular transmission (£53.8); Skeletal muscle relaxants (£9.9) |
|  | Soft-Tissue Disorders & Topical Pain Rel (£5.5) | Enzymes (£6.9); Rubefacients, topical NSAIDs, capsaicin, and poultices (£5.5) |
| Eye (£7.5) | Anti-Infective Eye Preparations (£5.6) | Antibacterials (£5.4); Antifungals (£321.8); Antivirals (£18.2) |
|  | Corti'roids & Other Anti-Inflamm.Preps. (£4.4) | Corticosteroids (£5.9); Other anti-inflammatory preparations (£3.6) |
|  | Mydriatics And Cycloplegics (£28.8) | No paragraphs |
|  | Treatment Of Glaucoma (£10.4) | No paragraphs |
|  | Local Anaesthetics (£3.9) | No paragraphs |
|  | Miscellaneous Ophthalmic Preparations (£4.7) | Tear deficiency, ocular lubricants, and astringents (£4.5); Ocular diagnostic and peri-operative preparations and photodynamic treatment (£17.9) |
| Ear, nose, and oropharynx (£6.1) | Drugs Acting On The Ear (£5.8) | Otitis externa (£6.0); Otitis media (£6.8); Removal of earwax (£2.4) |
|  | Drugs Acting On The Nose (£6.3) | Drugs used in nasal allergy (£5.5); Topical nasal decongestants (£29.4); Nasal preparations for infection (£2.8) |
|  | Drugs Acting On The Oropharynx (£5.6) | Drugs for oral ulceration and inflammation (£5.9); Oropharyngeal anti-infective drugs (£4.0); Lozenges and sprays (£3.0); Mouthwashes, gargles, and dentifrices (£4.0); Treatment of dry mouth^a^ (£9.7) |
| Skin (£7.9) | Vehicles & Emulsifying Agents (£4.3) | Vehicles (£4.3); Suitable quantities for prescribing (£NA); Excipients and sensitisation (£NA) |
|  | Management of Skin Conditions (£4.3) | Vehicles (£4.3); Suitable quantities for prescribing (£NA); Excipients and sensitisation (£NA) |
|  | Emollient & Barrier Preparations (£6.7) | Emollients (£6.9); Barrier preparations (£3.2) |
|  | Top Local Anaesthetics & Antipruritics (£6.2) | No paragraphs |
|  | Topical Corticosteroids (£4.9) | No paragraphs |
|  | Preparations For Eczema And Psoriasis (£41.9) | Preparations for eczema (£78.7); Preparations for psoriasis (£42.3); Drugs affecting the immune response (£35.3) |
|  | Acne and Rosacea (£14.7) | Topical preparations for acne (£14.4); Oral preparations for acne (£16.5); Topical preparations for rosacea (£36.0) |
|  | Preparations For Warts And Calluses (£10.2) | No paragraphs |
|  | Sunscreens And Camouflagers (£31.4) | Sunscreen preparations (£32.9); Camouflagers (£18.1) |
|  | Shampoo&Other Preps For Scalp&Hair Cond (£5.8) | No paragraphs |
|  | Anti-Infective Skin Preparations (£4.7) | Antibacterial preparations (£5.1); Antifungal preparations (£3.5); Antiviral preparations (£3.5); Parasiticidal preparations (£12.8); Preparations for minor cuts and abrasions (£4.7) |
|  | Skin Cleansers,Antiseptics & Desloughing (£8.6) | Alcohols and saline (£5.9); Chlorhexidine salts (£7.0); Cationic surfactants and soaps (£9.0); Iodine (£8.3); Phenolics (£7.1); Oxidisers and dyes (£14.2); Desloughing agents (£249.5) |
|  | Antiperspirants (£6.3) | No paragraphs |
|  | Wound Management Products (£29.8) | No paragraphs |
|  | Topical Circulatory Preparations (£4.9) | No paragraphs |
|  | Miscellaneous Topical Preparations (£137.3) | No paragraphs |
| Immunlogical products and vaccines (£9.1) | Vaccines And Antisera (£9.1) | No paragraphs |
|  | Immunoglobulins (£342.2) | Normal immunoglobulin (£441.5); Disease-specific immunoglobulins (£212.4); Anti-D (Rh0) immunoglobulin (£79.0) |
| Anaesthesia (£12.6) | General Anaesthesia (£12.6) | Intravenous anaesthetics (£79.3); Inhalational anaesthetics (£36.6); Antimuscarinic drugs (£21.7); Sedative and analgesic peri-operative drugs (£7.7); Neuromuscular blocking drugs (£NA); Drugs for reversal of neuromuscular blockade (£NA); Antagonists for central and respiratory depression (£42.6); Drugs for malignant hyperthermia (£NA) |
|  | Local Anaesthesia (£12.6) | No paragraphs |
| Other (£20.9) | Preparations used in diagnosis (£118.2) | No paragraphs |
|  | Other drugs and preparations (£28.0) | No paragraphs |
|  | Dressings (£21.0) | No paragraphs |
|  | Appliances (£13.4) | No paragraphs |
|  | Incontinence appliances (£25.7) | No paragraphs |
|  | Stoma appliances (£50.8) | No paragraphs |
| ^a^In these paragraphs the case mix of drugs types observed in CPRD differed substantially from that in the PCA. For these paragraphs, costs were based on the case mix of drugs observed in CPRD and unit costs of these drugs in PCA. | | |

Table S6. Distribution of women aged 55 to 79 years in England in 2013 by BMI category

| **BMI (kg/m^2^)** | **Percentage of women^a^** | **Number of women^b^** | **Mean measured BMI (kg/m^2^)^c^** |
| --- | --- | --- | --- |
| <18.5 | 1.7% | 114,257 | 18.9 |
| 18.5-19.9 | 4.1% | 273,554 | 20.2 |
| 20-22.4 | 14.2% | 940,219 | 22.5 |
| 22.5-24.9 | 22.6% | 1,497,925 | 25.1 |
| 25-27.4 | 20.3% | 1,346,245 | 27.6 |
| 27.5-29.9 | 14.1% | 930,615 | 30.1 |
| 30-34.9 | 16.0% | 1,061,099 | 33.8 |
| 35-39.9 | 4.6% | 302,367 | 39.1 |
| 40+ | 2.4% | 157,641 | 45.1 |
| BMI=body mass index  ^a^The percentage of the population in each category was estimated using the 2012 and 2013 Health Survey for England based on women aged 55 to 79 with self-reported height and weight.  ^b^Based on Office for National Statistics’ mid-2013 population estimates there were 6,623,923 women aged 55 to 79 in England. The number of women in each BMI category was estimated by multiplying the proportions in each category from the Health Survey for England to this total population estimate  ^c^Mean BMI within categories of self-reported BMI from the Health Surveys for England 2012 and 2013 | | | |

Table S7. Estimates of percentage increases in annual primary care consultation costs under sensitivity analyses

|  | **Body Mass Index (kg/m^2^)** | | | | | | | |  |
| --- | --- | --- | --- | --- | --- | --- | --- | --- | --- |
|  | **18.5-19.9** | **20-22.4**  **(reference)** | **22.5-24.9** | **25-27.4** | **27.5-29.9** | **30-34.9** | **35-39.9** | **40+** | **Trend^a^** |
| Base case analysis | 0.9  (-3.8, 5.8) | 0.0  (-2.0, 2.1) | 2.7  (1.2, 4.3) | 9.1  (7.3, 10.9) | 17.5  (15.1, 19.8) | 30.7  (27.8, 33.5) | 48.9  (42.9, 55.3) | 64.5  (53.5, 76.3) | 5.2  (4.8, 5.6) |
| Including pre-existing cancer | 0.8  (-3.8, 5.6) | 0.0  (-2.0, 2.1) | 2.6  (1.1, 4.2) | 9.2  (7.4, 11.0) | 17.4  (15.1, 19.7) | 30.9  (28.1, 33.8) | 48.9  (42.9, 55.1) | 64.1  (53.2, 75.7) | 5.2  (4.8, 5.6) |
| Including all years of follow-up | 2.9  (-1.3, 7.4) | 0.0  (-1.8, 1.8) | 2.9  (1.5, 4.3) | 9.5  (7.9, 11.1) | 18.1  (16.0, 20.2) | 30.7  (28.2, 33.3) | 49.6  (44.3, 55.1) | 60.8  (51.9, 70.4) | 5.1  (4.8, 5.5) |
| Excluding BMI >50 kg/m^2^ | 0.9  (-3.8, 5.8) | 0.0  (-2.0, 2.1) | 2.7  (1.2, 4.3) | 9.1  (7.3, 10.9) | 17.5  (15.1, 19.8) | 30.6  (27.8, 33.5) | 48.9  (42.9, 55.3) | 62.1  (51.2, 73.9) | 5.2  (4.8, 5.6) |
| Never smokers only | -0.9  (-7.4, 6.2) | 0.0  (-2.7, 2.8) | 4.7  (2.6, 6.9) | 10.0  (7.5, 12.5) | 18.6  (15.1, 22.2) | 30.7  (26.7, 34.7) | 54.6  (45.5, 64.4) | 66.8  (49.6, 85.9) | 5.3  (4.7, 5.9) |
| Excluding pre-existing heart disease and stroke | 0.0  (-4.7, 4.9) | 0.0  (-2.1, 2.1) | 2.9  (1.4, 4.5) | 9.2  (7.4, 11.0) | 17.4  (15.0, 19.8) | 30.8  (27.9, 33.8) | 47.6  (41.1, 54.3) | 61.7  (50.2, 74.0) | 5.1  (4.7, 5.5) |
| Mean measured BMI from HSE | - | - | - | - | - | - | - | - | 5.1  (4.7, 5.5) |
| In each model, adjustments were made for participant’s age, region of recruitment, deprivation, educational qualifications, parity, age at first birth, smoking, alcohol intake, financial year and proportion of year with contributed data.  ^a^Percentage change in annual costs per 2 kg/m^2^ increase in BMI (BMI ≥20 kg/m^2^) | | | | | | | | | |

Table S8. Estimates of percentage increases in annual prescription costs under sensitivity analyses

|  | **Body Mass Index (kg/m^2^)** | | | | | | | |  |
| --- | --- | --- | --- | --- | --- | --- | --- | --- | --- |
|  | **18.5-19.9** | **20-22.4**  **(reference)** | **22.5-24.9** | **25-27.4** | **27.5-29.9** | **30-34.9** | **35-39.9** | **40+** | **Trend^a^** |
| Base case analysis | 12.9  (2.5, 24.3) | 0.0  (-4.0, 4.2) | 2.6  (-0.6, 5.8) | 16.4  (12.7, 20.2) | 33.1  (26.8, 39.7) | 62.8  (56.7, 69.1) | 103.0  (90.1, 116.9) | 158.7  (134.1, 185.9) | 9.9  (9.2, 10.6) |
| Including pre-existing cancer | 12.8  (2.6, 24.0) | 0.0  (-4.0, 4.2) | 2.1  (-1.0, 5.3) | 16.6  (12.6, 20.7) | 33.1  (27.0, 39.6) | 63.7  (57.2, 70.5) | 102.3  (89.5, 115.9) | 156.4  (132.3, 183.1) | 9.9  (9.2, 10.6) |
| Including all years of follow-up | 16.5  (6.1, 27.9) | 0.0  (-3.9, 4.1) | 1.1  (-1.9, 4.1) | 12.8  (9.4, 16.4) | 30.2  (24.5, 36.1) | 57.3  (51.7, 63.1) | 96.5  (84.7, 109.1) | 142.8  (120.7, 167.1) | 9.4  (8.7, 10.0) |
| Excluding BMI >50 kg/m^2^ | 12.9  (2.5, 24.3) | 0.0  (-4.0, 4.2) | 2.6  (-0.6, 5.8) | 16.3  (12.7, 20.1) | 33.1  (26.8, 39.7) | 62.8  (56.7, 69.1) | 103.1  (90.1, 116.9) | 153.8  (129.1, 181.3) | 10.0  (9.3, 10.7) |
| Never smokers only | -2.1  (-13.8, 11.2) | 0.0  (-6.2, 6.7) | 4.1  (-0.5, 8.8) | 17.9  (12.3, 23.7) | 37.4  (29.8, 45.5) | 63.1  (54.1, 72.5) | 120.1  (99.4, 142.9) | 177.9  (137.2, 225.5) | 10.5  (9.5, 11.6) |
| Excluding pre-existing heart disease and stroke | 12.2  (1.5, 24.0) | 0.0  (-4.2, 4.4) | 3.0  (-0.3, 6.3) | 15.8  (12.0, 19.7) | 31.9  (25.2, 39.0) | 60.8  (54.5, 67.3) | 95.4  (82.2, 109.5) | 148.8  (124.2, 176.1) | 9.5  (8.8, 10.2) |
| Mean measured BMI from HSE | - | - | - | - | - | - | - | - | 9.6  (8.9, 10.3) |
| In each model, adjustments were made for participant’s age, region of recruitment, deprivation, educational qualifications, parity, age at first birth, smoking, alcohol intake, financial year and proportion of year with contributed data.  ^a^Percentage change in annual costs per 2 kg/m^2^ increase in BMI (BMI ≥20 kg/m^2^) | | | | | | | | | |

Table S9. Estimates of percentage increases in annual diagnostic test costs under sensitivity analyses

|  | **Body Mass Index (kg/m^2^)** | | | | | | | |  |
| --- | --- | --- | --- | --- | --- | --- | --- | --- | --- |
|  | **18.5-19.9** | **20-22.4**  **(reference)** | **22.5-24.9** | **25-27.4** | **27.5-29.9** | **30-34.9** | **35-39.9** | **40+** | **Trend^a^** |
| Base case analysis | 6.7  (-6.0, 21.1) | 0.0  (-5.4, 5.8) | -2.0  (-6.0, 2.1) | 0.4  (-4.2, 5.2) | -1.8  (-7.4, 4.1) | -2.6  (-8.6, 3.9) | -2.4  (-13.1, 9.6) | 14.4  (-8.0, 42.1) | 0.0  (-1.0, 1.1) |
| Including pre-existing cancer | 5.6  (-6.8, 19.7) | 0.0  (-5.4, 5.7) | -2.2  (-6.1, 1.8) | 0.2  (-4.3, 5.0) | -1.6  (-7.2, 4.2) | -2.9  (-8.9, 3.5) | -3.1  (-13.7, 8.7) | 13.6  (-8.4, 40.9) | 0.0  (-1.1, 1.1) |
| Including all years of follow-up | 6.3  (-5.4, 19.5) | 0.0  (-4.8, 5.1) | -1.4  (-5.0, 2.4) | 1.4  (-2.9, 5.9) | -1.2  (-6.3, 4.2) | -2.0  (-7.6, 3.9) | -2.4  (-12.2, 8.4) | 11.3  (-9.4, 36.9) | 0.0  (-1.0, 1.0) |
| Excluding BMI >50 kg/m^2^ | 6.7  (-6.0, 21.2) | 0.0  (-5.4, 5.8) | -2.0  (-6.0, 2.0) | 0.4  (-4.2, 5.2) | -1.8  (-7.4, 4.0) | -2.6  (-8.6, 3.9) | -2.4  (-13.1, 9.6) | 12.6  (-10.1, 40.9) | -0.1  (-1.1, 1.1) |
| Never smokers only | 2.5  (-14.7, 23.2) | 0.0  (-7.0, 7.6) | -1.4  (-6.8, 4.4) | 0.4  (-5.9, 7.1) | -0.3  (-8.6, 8.8) | -1.2  (-9.8, 8.1) | 0.2  (-14.8, 17.8) | 17.6  (-17.7, 68.1) | 0.3  (-1.2, 1.9) |
| Excluding pre-existing heart disease and stroke | 8.0  (-5.1, 22.8) | 0.0  (-5.5, 5.8) | -1.4  (-5.5, 2.7) | 1.0  (-3.7, 6.0) | -0.5  (-6.3, 5.6) | -3.0  (-9.2, 3.6) | -6.3  (-16.9, 5.7) | 10.9  (-11.8, 39.4) | -0.2  (-1.3, 0.9) |
| Mean measured BMI from HSE | - | - | - | - | - | - | - | - | 0.0  (-1.1, 1.0) |
| In each model, adjustments were made for participant’s age, region of recruitment, deprivation, educational qualifications, parity, age at first birth, smoking, alcohol intake, financial year and proportion of year with contributed data.  ^a^Percentage change in annual costs per 2 kg/m^2^ increase in BMI (BMI ≥20 kg/m^2^) | | | | | | | | | |

Table S10. Annual prescription costs attributed to overweight and obesity among women aged 55 to 79 years in England, by therapeutic use

| **BNF chapter/section** | **Total annual prescription costs (£million)** | **Costs attributed to overweight and obesity (BMI≥25kg/m^2^)** | |
| --- | --- | --- | --- |
|  |  | **Annual attributed prescription costs (£million) (99% CI)** | **Proportion costs attributed**  **(%) (99% CI)** |
| Gastrointestinal system [01] | 119 | 13 (9, 17) | 11 (8, 14) |
| Cardiovascular system [02] | 248 | 73 (68, 78) | 30 (28, 31) |
| Diuretics [0202] | 16 | 6 (6, 6) | 37 (35, 39) |
| Beta-adrenoceptor blocking drugs [0204] | 16 | 4 (3, 4) | 24 (21, 26) |
| Hypertension and heart failure [0205] | 38 | 14 (13, 15) | 36 (34, 38) |
| Nitrates, calcium-channel blockers, and other antianginal drugs [0206] | 43 | 12 (11, 13) | 28 (25, 30) |
| Anticoagulants and protamine [0208] | 54 | 17 (14, 20) | 31 (25, 36) |
| Antiplatelet drugs [0209] | 16 | 4 (4, 4) | 26 (23, 28) |
| Lipid-regulating drugs [0212] | 58 | 15 (14, 16) | 26 (24, 27) |
| Other cardiovascular system | 7 | 2 (1, 3) | 28 (19, 36) |
| Respiratory system [03] | 222 | 26 (15, 41) | 12 (7, 17) |
| Analgesics [0407] | 181 | 51 (46, 56) | 28 (26, 30) |
| Central nervous system (excl. analgesics) [04] | 163 | 20 (13, 28) | 12 (8, 17) |
| Infections [05] | 46 | 5 (2, 7) | 10 (5, 15) |
| Drugs in Diabetes [0601] | 153 | 102 (93, 113) | 67 (63, 70) |
| Endocrine system (excl. diabetes) [06] | 98 | 4 (0, 7) | 4 (0, 8) |
| Obstetrics, gynaecology, and urinary-tract disorders [07] | 65 | 13 (10, 16) | 20 (16, 24) |
| Malignant disease and immunosuppression [08] | 164 | 9 (0, 20) | 5 (0, 12) |
| Nutrition and blood [09] | 153 | 0 (0, 0) | 0 (0, 0) |
| Musculoskeletal and joint diseases [10] | 44 | 9 (8, 11) | 20 (17, 24) |
| Eye [11] | 46 | 0 (0, 0) | 0 (0, 0) |
| Ear, nose, and oropharynx [12] | 18 | 0 (0, 1) | 3 (0, 7) |
| Skin [13] | 47 | 8 (6, 10) | 17 (13, 21) |
| Immunological products and vaccines [14] | 13 | 0 (0, 0) | 1 (0, 3) |
| Anaesthesia [15] | 4 | 0 (0, 1) | 9 (5, 14) |
| Dressings and appliances | 132 | 48 (41, 56) | 36 (32, 41) |
| Prescriptions issued were categorised by therapeutic use (defined according to British National Formulary chapters and sections). Overweight and obesity is defined as a body mass index (BMI) ≥ 25 kg/m^2^. Estimates were derived by combining estimates of annual costs per person for each therapeutic use category (Figure S4) and estimates of the number of women aged 55 to 79 in England by self-reported BMI category (Table S6). | | | |

Table S11. Search terms used to identify relevant evidence before the study

| Number | MEDLINE | EMBASE |
| --- | --- | --- |
| 1 | economics/ | health economics/ |
| 2 | exp “costs and cost analysis” / | exp health care cost/ |
| 3 | exp economics, medical/ | exp fee/ |
| 4 | exp economics, hospital/ | budget/ |
| 5 | economics, nursing/ | funding/ |
| 6 | economics, pharmaceutical/ | cost*.ti |
| 7 | exp “fees and charges”/ | economic*.ti |
| 8 | cost*.ti | (expenditure* or charge*).ti |
| 9 | economic*.ti | cost of illness/ |
| 10 | (expenditure* or charge*).ti | 1 or 2 or 3 or 4 or 5 or 6 or 7 or 8 or 9 |
| 11 | exp “budgets”/ | exp obesity/ |
| 12 | cost of illness/ | (obesity or obese).ti |
| 13 | 1 or 2 or 3 or 4 or 5 or 6 or 7 or 8 or 9 or 10 or 11 or 12 | (overweight or over-weight or over weight).ti |
| 14 | exp obesity/ | (bmi or body mass index or body mass).ti |
| 15 | exp overweight/ | 11 or 12 or 13 or 14 |
| 16 | (obesity or obese).ti | 10 and 15 |
| 17 | (overweight or over-weight or over weight).ti |  |
| 18 | (bmi or body mass index or body mass).ti |  |
| 19 | 14 or 15 or 16 or 17 or 18 |  |
| 20 | 13 and 19 |  |
| Figure S1. Trends in annual total consultation costs per 2 kg/m^2^ increase in BMI for BMI 20 kg/m^2^ or above, by category of women  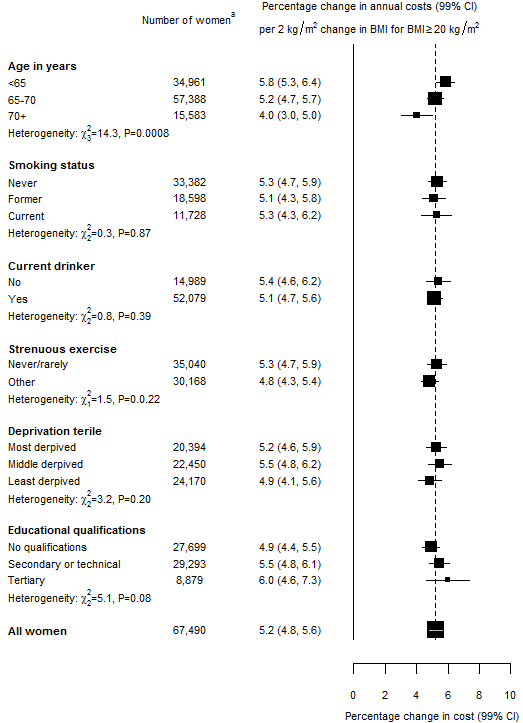  BMI= body mass index. Estimation adjusted for age, region of recruitment, deprivation, educational qualifications, parity, age at birth of first child, smoking, alcohol intake, financial year, and proportion of the year with contributed data, as appropriate. The area of each square is inversely proportional to the variance. All estimates of percentage change in annual costs have p-values<0.001. Participants with missing data on particular characteristics were excluded; the proportion of missing data is less than 3% for all characteristics except for smoking status (5%).  ^a^Numbers based on women with BMI≥20 kg/m^2^ only. | | |

| Figure S2. Trends in annual total prescription costs per 2 kg/m^2^ increase in BMI for BMI 20 kg/m^2^ or above, by category of women  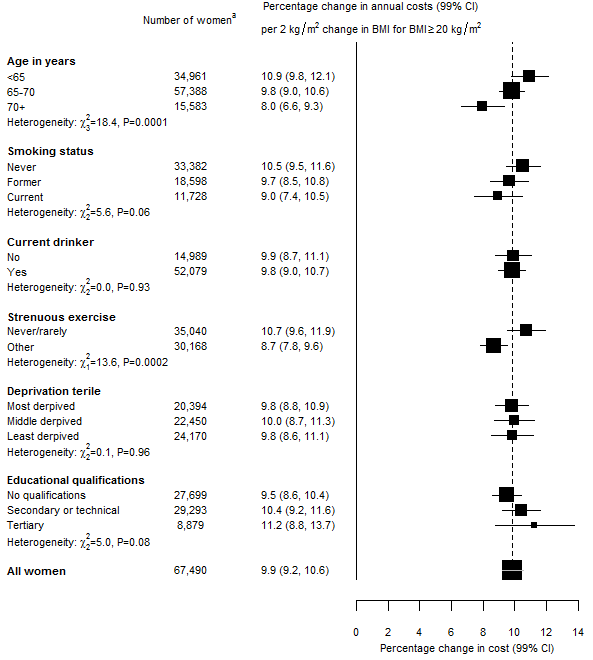  BMI= body mass index. Estimation adjusted for age, region of recruitment, deprivation, educational qualifications, parity, age at birth of first child, smoking, alcohol intake, financial year, and proportion of the year with contributed data, as appropriate. The area of each square is inversely proportional to the variance. All estimates of percentage change in annual costs have p-values<0.001. Participants with missing data on particular characteristics were excluded; the proportion of missing data is less than 3% for all characteristics except for smoking status (5%).  ^a^Numbers based on women with BMI≥20 kg/m^2^ only. |
| --- |

| Figure S3. Trends in annual total diagnostic test costs per 2 kg/m2 increase in BMI for BMI 20 kg/m2 or above, by category of women  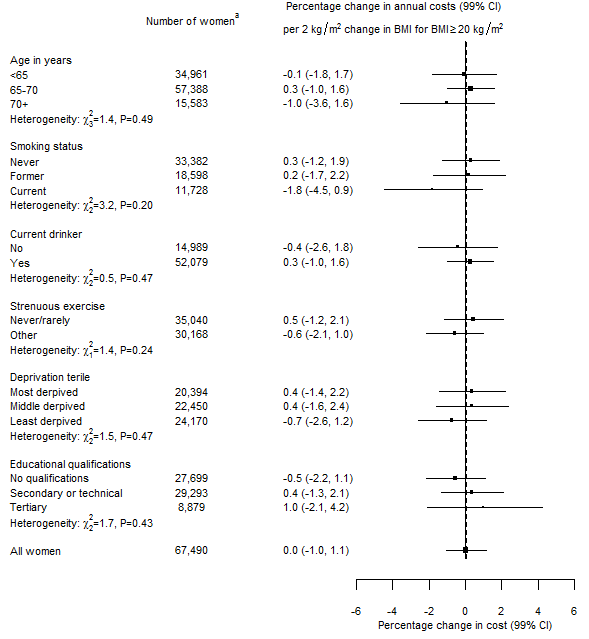  BMI= body mass index. Estimation adjusted for age, region of recruitment, deprivation, educational qualifications, parity, age at birth of first child, smoking, alcohol intake, financial year, and proportion of the year with contributed data, as appropriate. The area of each square is inversely proportional to the variance. All estimates of percentage change in annual costs have p-values<0.001. Participants with missing data on particular characteristics were excluded; the proportion of missing data is less than 3% for all characteristics except for smoking status (5%).  ^a^Numbers based on women with BMI≥20 kg/m^2^ only. |
| --- |

| Figure S4. Annual prescription costs per person by body mass index and therapeutic use  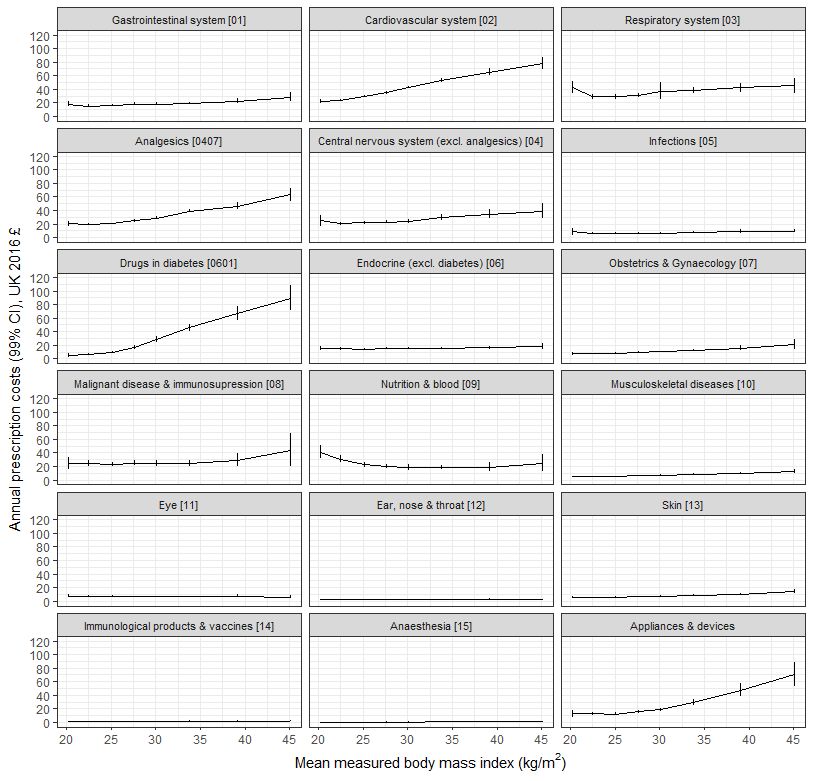  Estimates derived from GLM log-link Poisson variance regression models with separate models were fitted for each category of therapeutic use, adjusted for age, region of recruitment, deprivation, educational qualifications, parity, age at first birth, smoking, financial year, and the proportion of the year with contributed data. Annual costs are plotted against mean measured BMI within categories of self-reported BMI from the combined 2012 and 2013 Health Surveys for England (Table S6). |
| --- |

# Statistical Appendix

**Costing consultations**

Where available, costs per consultation were taken from the Unit Costs of Health and Social Care 2016. For nurses these costs include salaries, salary on-costs, qualifications, overheads, and capital overheads. For GPs, they include net remuneration, practice expenses (administrative and clerical staff, office and general business, premises, other), pre-registration and post-graduate medical education, and capital costs. For other healthcare professionals costs were often taken from the NHS Reference Cost schedule 2015/16. NHS Reference Costs are calculated on a full absorption basis, including direct costs (e.g. consultants, nurses), indirect costs (e.g. laundry, lighting), and overheads (e.g. administration costs).

Records generated by staff with unknown roles (7%) were excluded. In a sensitivity analysis, staff roles, where unknown, were imputed using the relative frequency of each staff role by category of consultation type among observed cases. Imputation had no material impact on the results. Duplicate records, defined as more than one consultation of a particular type (e.g. face-to-face surgery consultation) with a particular category of staff (e.g. GP), were excluded (5% of all consultations).

**Costing prescriptions**

The prescription analysis contains records of the number of items and net ingredient costs (NIC) of all prescriptions dispensed in the community in England. The data is categorised according to the British National Formulary (BNF) code, or, for items not included in the BNF chapters (01-15), pseudo-BNF codes, which contain mostly appliances and devices. The NIC is the basic price of the medicine, that is, the price listed in the National Drug Tariff, Part VIII or IX (<http://www.nhsbsa.nhs.uk/PrescriptionServices/4940.aspx>), or for drugs not listed in the Drug Tariff, the list price published by the manufacturer, wholesale, or supplier. The NIC represents the cost of the drug before any discounts are applied, does not include any dispensing costs or fees, and does not adjust for income obtained where a prescription charge is paid at the time the prescription is dispensed or where the patient has purchased a pre-payment certificate. Duplicate prescriptions within a single consultation were excluded (1%).

As of November 2016, the BNF contained 15 chapters (e.g. chapter 06: Endocrine system), each describing medications relating to a system of the body or aspect of medical care, within which there are a total of 113 sections (e.g. section 0601: Drugs in diabetes), and 226 paragraphs (e.g. paragraph 060101: Insulins). There are also five pseudo-BNF chapters (defined by the NHS Business Authority), consisting mostly of devices and appliances, for items not in the standard BNF.

**Costing tests**

We included laboratory tests, diagnostic tests, and diagnostic imaging. We additionally excluded a number of diagnostic tests which are likely to be performed as part of a routine consultation (0.2%): pulmonary function tests, ambulatory blood pressure, skin prick test, forced expiration (FEV) volume in 1 sec, forced vital capacity (FVC), FEV1 / FVC, transfer coefficient for carbon monoxide, airway reversibility, total lung capacity, intraocular pressure left, and intraocular pressure right. We also excluded diagnostic tests that coincided with outpatient appointments (0.04%). Costs were taken from the NHS Reference Cost schedule 2015/16, which were calculated on a full absorption basis. Duplicate records of a test within a single consultation were excluded (1%).

**Statistical modelling**

Statistical model selection was performed within the generalized linear model (GLM) framework.^1^ Common specification tests were used to select appropriate GLMs for the following total annual costs and only positive annual costs for the following outcomes: all consultations, all prescriptions, and prescriptions by category of therapeutic use.^2^ We compared the selected models with other commonly used models (linear regression, two-part log-gamma models) in terms of metrics of fit (mean error, mean absolute error, and root mean squared error) overall and by BMI category at baseline and deciles of predicted cost (from linear regression), and in terms of predicted marginal effects by BMI category. We found that single-equation Poisson regression models performed as well as two-part log-gamma models for annual costs and produced very similar marginal effects. All models were estimated using cluster-robust standard errors to account for the lack of independence between observations of annual costs for the same participant in different years. Relative rates of admission and relative costs are presented in tables and figures with group-specific 99% confidence intervals, which can be compared between any pair of categories, even if neither is the reference category.^3,4^

In all models, adjustments were made for: age (in five-year bands), region of recruitment (nine regions corresponding to the areas covered by the cancer registries in England), quintiles of socioeconomic status based on the Townsend deprivation index,[^15^](#_ENREF_15) parity (nulliparous, 1, 2, ≥3), age at birth of first child (<25, 25-29, ≥30 years), smoking (never, past, current), alcohol intake (rarely/never, <7 units/week, ≥7 units/week), educational qualifications (no qualifications, secondary, technical, tertiary), financial year, and the proportion of each year with contributed data. The covariates included in the model were informed by a systematic literature review of cost studies and other identified epidemiologic studies. Demographic and physical characteristics were based on those reported by participants at recruitment into the Million Women Study. We also examined the effects of addition of each baseline variables to a minimally adjusted model consisting of BMI category, age group, region and HES year.

**Mean measured BMI within categories of self-reported BMI**

The Health Survey for England (HSE) is an annual survey designed to collect information on the health and health-related behaviour of people living in private households in England. We used the 2012 and 2013 HSE data in this analysis. The 2012 and 2013 data in HSE had information on 21,313 individuals (10,333 from 2012 and 10,980 from 2013). After restricting to women aged 50-64 (the age of participants at reporting of height and weight in the MWS), the combined sample consisted of 2,087 women. Measured height and/or weight was missing for 315 (15%) of them. BMI was categorised using self-reported BMI as in analysis (see above) and means of measured BMIs within each category of self-reported BMI were derived (Table S6).

**Extrapolation of costs to women aged 55 to 79 in England**

Costs attributable to each category of overweight and obesity and for overweight and obesity combined (i.e. excess weight; BMI ≥25 kg/m^2^) were estimated for all women aged 55 to 79 years in England in 2013, for total consultation costs, total prescription costs, and prescription costs by category of therapeutic use. Differences in standardised mean costs between each overweight and obesity category and healthy weight (BMI 20 to <25 kg/m^2^) were calculated and then multiplied by the number of women aged 55 to 79 years in England within the respective BMI category (Table S6). These numbers were estimated by applying information on the distribution of self-reported BMI categories from the 2012 and 2013 Health Surveys for England to the ONS mid-2013 population estimate of the total number of women aged 55 to 79 years in England: 6,623,923.^5^

Confidence intervals around each of these outcomes were generated through a simulation process. 10,000 sets of correlated model parameters were generated from the model equations using the Cholesky decomposition. For each parameter set, the estimates of overweight and obesity attributable costs were estimated as describe above. 99% confidence intervals were calculated taking the 0.5^th^ and 99.5^th^ percentiles of the resulting distribution as the confidence limits.

**Identification of diabetes**

*Identification of diabetes*

If a women self-reported diabetes at recruitment into the MWS or had a hospital record containing an ICD-10 code for diabetes (E10-E14 excluding E11) in any diagnostic position, then she was considered to have diabetes. Diabetes was also identified using CPRD data, taking three separate approaches. First, a woman was considered to have diabetes is she had any READ version 2 code in the list below. This list was constructed from all C10F codes and any codes used for identification of type-2 diabetes in recent publications.^6-10^ Using all codes as opposed to only C10F codes as sometimes recommended^6^ identified 8.8% of women as having diabetes compared to 8.1% using only C10F codes. Diabetes was also considered present if a participant was prescribed any drug used in diabetes (BNF section 0601). This led to very similar identification as when using specific product codes listed in previous publications.^7-9^ It includes metformin which can be used in pre-diabetes. Finally, again following the precedence of previous publications,^6^ diabetes was identified from the results of HbA1c (entity type codes: 1, 97) or glucose tolerance (entity codes: 213, 274) tests. Diabetes is indicated by a hbA1c test result of ≥6.5% or ≥48 mmol/mol. For those tests which are known to be fasting glucose (26%), diabetes was indicated by a value of ≥7.0%. Where it was not stated whether the glucose was fasting or not, a conservative threshold of ≥11.1% was used. A women was only considered to have diabetes if she had more than one test result (of any type) indicative of diabetes. The date of onset of diabetes was taken as the earliest date on which diabetes was identified using any of the strategies defined above. Diabetes if considered present in each person-year if the person has any evidence of diabetes in that year or any previous year.

| **Diabetes code list (READ version 2 codes)** |
| --- |
| C100112, C10F.00, C10FJ00, 66A4.00, C109.00, C109.11, 66A3.00, C109700, C10FC00, C10F500, C100111, C109600, C109.12, C109G11, C109012, C109.13, C109J12, C109J00, C10FM00, C10FB00, C10F600, C10F000, C10N.00, C10F.11, C109711, C109G00, C109C12, ZC2CA00, C10FQ00, C10F700, C10FL00, 66AV.00, C109900, C10FN00, C107200, C10A100, C10F200, C10FK00, C109400, C104100, C10FH00, C109K00, C10D.00, C109J11, C10FF00, C106100, C10F811, C109500, C109H00, C105100, C109612, C102100, C10F311, C10C.00, C109D00, C109E12, C10FE00, C109B00, C109712, C109212, C109512, C10C.11, C10FD00, C10F711, C10F100, C109B11, C109H11, C10F900, C109E11, C10F400, C10F611, C109G12, C109011, C109100, C10FB11, L180600, C109A11, C10G.00, C10FP00, C109000, C10F911, C109F00, C10F800, C101100, C109F11, C109411, 66AO.00, C109200, C109D11, C107400, C10F011, C109611, C10FG00, C103y00, C109C00, C109111, C10D.11, C109F12, C10FL11, C109D12, C109511, C109300, C10FA00, C107100, C10FR00, C10z100, C109C11, C10FJ11, C10F300, C109412, C109H12, C109211, C109E00, C109112, C109A00, 66Ao.00, C10FM11, C10F411, C10N100, C10FE11, C10FA11, C10FS00, C10ER00, C10F211, C10FD11, C10F111, 66At100, C10FC11, 66At111, C10FG11, C10F511, C10FF11, C109912, C10FP11, C10FN11, C10FK11 |

**Statistical Appendix References**

1. Blough D, Maden C, Hombrook M. Modeling risk using generalized linear models. *J Health Econ* 1999; **18**(2): 153-71.

2. Manning WG, Mullahy J. Estimating log models: to transform or not to transform? *J Health Econ* 2001; **20**(4): 461-94.

3. Easton DF, Peto J, Babiker AG. Floating absolute risk: an alternative to relative risk in survival and case-control analysis avoiding an arbitrary reference group. *Statistics in medicine* 1991; **10**(7): 1025-35.

4. Plummer M. Improved estimates of floating absolute risk. *Statistics in medicine* 2004; **23**(1): 93-104.

5. Office for National Statistics. Population Estimates for UK, England and Wales, Scotland and Northern Ireland, Mid-2013. 2014. http://www.ons.gov.uk/ons/publications/re-reference-tables.html?edition=tcm%3A77-322718 (accessed 28 April 2015).

6. Eastwood SV, Mathur R, Atkinson M, et al. Algorithms for the Capture and Adjudication of Prevalent and Incident Diabetes in UK Biobank. *PLoS ONE* 2016; **11**(9): e0162388.

7. van Brunt K, Curtis B, Ivanyi T, et al. Basal-bolus Therapy in Patients with Type 2 Diabetes Mellitus in the UK: Patient Characteristics, Treatment Patterns and the Effect of Switching to Premixed Insulin. *Diabetes Ther* 2016; **7**(4): 793-807.

8. Kontopantelis E, Reeves D, Valderas JM, Campbell S, Doran T. Recorded quality of primary care for patients with diabetes in England before and after the introduction of a financial incentive scheme: a longitudinal observational study. *BMJ Qual Saf* 2013; **22**(1): 53-64.

9. Wright AK, Kontopantelis E, Emsley R, et al. Life Expectancy and Cause-Specific Mortality in Type 2 Diabetes: A Population-Based Cohort Study Quantifying Relationships in Ethnic Subgroups. *Diabetes Care* 2017; **40**(3): 338-45.

10. Zhong V, Juhaeri J, Cole S, et al. Recent HbA1C level and risk of hypoglycemia hospitalization in adults with type 1 and type 2 diabetes: a nested case-control study. *Under review* 2017.
